# Nudging consumers about the issue of microplastics: an experimental auction study on valuation for sustainable food packaging

**DOI:** 10.1038/s41598-024-69962-8

**Published:** 2024-08-16

**Authors:** László Bendegúz Nagy, Rodolfo M. Nayga, Ágoston Temesi

**Affiliations:** 1 Hungarian University of Agriculture and Life Sciences, Villányi Str. 29-43, Budapest, 1118 Hungary; 2 Texas A&M University, College Station, Texas USA; 3 Korea University, Seoul, South Korea

**Keywords:** Biodegradable packaging, BDM, Willingness to pay, Trust, Pasta, Product environmental footprint, Sustainability, Psychology and behaviour, Environmental economics, Environmental impact

## Abstract

Plastic, integral to food packaging since the 1950s, has become a global environmental concern due to its contribution to microplastic pollution. Microplastics harm ecosystems, impacting wildlife and human health. Amid increasing focus on sustainability, global initiatives target sustainable production and consumption, but consumers struggle to verify product claims, leading to potential greenwashing, particularly in the food industry. We conducted an experiment focusing on pasta products with varied packaging and labeling attributes. Findings suggest that consumers are willing to pay more for products with both biodegradable packaging and Product Environmental Footprint (PEF) labels, indicating heightened trust and perceived sustainability. Information about microplastics’ adverse environmental effects influenced consumer valuation, particularly among females, higher-income individuals, and those with stronger environmental concerns.

## Introduction

Since the 1950s, plastic has become ubiquitous in food packaging, contributing significantly to the global plastic production, as reported by the United Nations Environment Programme (UNEP) in 2018^1^. However, the widespread use of single-use plastic packaging has led to severe environmental consequences, primarily due to its propensity to break down into minuscule particles known as microplastics, as highlighted by Hale et al.^2^.

Food packaging stands prominently as a primary source of microplastics, constituting a pervasive environmental concern^3^. The lifecycle of plastic packaging culminates in its disposal, often ending up as non-biodegradable waste^4^. Over time, these discarded materials undergo fragmentation, breaking down into minuscule particles known as microplastics. Microplastics infiltrate various ecosystems, including marine environments^5^, terrestrial soils, and even the atmosphere^6^, and hence can inflict substantial harm on both wildlife and human health, with far-reaching consequences^7^.

Microplastics have been detected in many food products, like beer^8^, tea^9^, honey^10^ and fish^11^, showing an emerging concern for public health and food safety, as consuming microplastics can negatively influence digestive, respiratory and circulatory systems of the human body^12^.

Biodegradable plastics present a notable improvement over traditional plastic packaging for several reasons. Unlike conventional plastics that persist in the environment for centuries, biodegradable plastics have the capability to break down into organic materials when exposed to specific conditions^13^. This characteristic significantly reduces their environmental footprint, curbing the accumulation of non-degradable waste in landfills and ecosystems^14^. Furthermore, the decomposition of biodegradable plastics generates less harm to wildlife and marine ecosystems^12^. Overall, their capacity to transition into organic matter offers a more sustainable and responsible solution, aligning with the pressing need to reduce plastic-related environmental degradation and promote a cleaner, healthier planet^15^.

In recent years, sustainability has taken center stage in the food industry’s agenda. International policies now emphasize the ecological impact of large-scale food production, as these systems contribute significantly to global carbon dioxide emissions^16^, aligning with the United Nations’ 2030 Agenda for Sustainable Development, which includes a specific goal: “Ensure sustainable consumption and production patterns”^17^. This goal aims to reduce waste generation by 2030 and encourage companies to report their sustainability performance (Target 12.5 and 12.6, respectively). To support these objectives, the European Union has introduced the Farm to Fork strategy as part of the European Green Deal, designed to facilitate the transition towards more environmentally friendly practices in European agriculture and the food industry^18^.

While both companies and consumers now recognize the need for more sustainable products and consumption habits, consumers often find it challenging to assess the true environmental impact of the food products they purchase. Their primary source of information for evaluating a product’s sustainability comes from labels provided by producers or retailers, creating an information imbalance that turns sustainability claims into “credence attributes”—qualities that consumers cannot directly verify^19^. Unfortunately, this information gap also opens the door to “greenwashing,” where companies with poor environmental performance present themselves in a positive light^20^. Greenwashing is particularly prevalent in the packaging and labeling of food products^21^.

To combat greenwashing and provide consumers with more accurate information, the European Commission has introduced the Product Environmental Footprint (PEF) methodology, designed to uniformly assess a product’s environmental impact throughout its entire lifecycle. The PEF methodology is applicable to various consumer products, including food items, and holds significant potential for informing consumers about the sustainability of their purchases.

While the PEF methodology is a relatively recent development, limited research has explored consumer acceptance of this new labeling system. A report by the Ipsos consortium for the European Commission found that communicating PEF scores guides consumers toward more environmentally friendly choices^22^. In contrast, a choice-based experiment conducted by Limnios et al.^23^ revealed that consumers placed little value on PEF scores, largely due to their limited knowledge of the concept. To our knowledge, no prior field research has investigated consumers’ willingness to pay (WTP) for PEF-labeled products.

As pointed out by Steenis et al.^24^, the sustainability of a product depends on both its content and packaging, and consumers perceive it as deceptive when only one of these attributes (either the packaging or the product content) is sustainable. Although organic policies do not regulate the type of packaging for organic food products, there is limited research on the relationship between organic food and packaging^25^.

Assessing the actual sustainability of packaging is a complex task for consumers^26^. In addition to the challenge of assessment, consumers often lack knowledge about the environmental friendliness of packaging materials^27^. Providing additional information to consumers can assist in their decision-making process. For instance, research by Van Asselt et al.^28^ revealed that negative information about plastic packaging decreased consumers’ willingness to pay for a product. According to Wensing et al.^29^, green nudges can increase WTP, but nudges are only effective if they match consumers’ cognitive style. In some cases, consumers rely on the appearance of packaging rather than communicated information, posing a risk of misleading practices in the food industry^25^. However, despite the growing interest in environmentally friendly packaging, Ketelsen et al.^25^ found no field studies on consumers’ attitudes toward sustainable packaging in their review.

Given the importance of sustainable labeling and the urgency to reduce plastic pollution, this study aims to contribute to the existing literature on environmentally friendly consumer behavior by investigating the willingness to pay for sustainable food packaging and the influence of information regarding the harmful effects of microplastics. Specifically, we seek to identify key factors that impact consumers’ valuation decisions, particularly focusing on the role of gender, education level, income, and environmental consciousness.

Our primary research question explores the extent to which information on the detrimental effects of microplastics influences consumers’ willingness to pay for environmentally friendly products. Subsequently, we investigate how individual characteristics, such as gender, education level, income, and environmental consciousness, shape this influence. Additionally, we examine consumers’ valuation for biodegradable packaging and the PEF logo and explore whether their combined presence yields higher WTP for sustainable food packaging.

The findings of this study hold valuable insights for various stakeholders, including policymakers, marketers, and environmental advocates. Policymakers can use the information to design more effective regulations and initiatives aimed at promoting sustainable consumer choices, while marketers can tailor their strategies based on a deeper understanding of how individual characteristics influence consumer preferences for environmentally friendly products. Additionally, environmental advocates can leverage these insights to enhance their communication strategies and encourage more widespread adoption of sustainable packaging practices. Ultimately, the study contributes to the broader goal of fostering environmentally conscious consumer behavior and advancing sustainability in the marketplace.

## Methods and design

**Figure 1:**
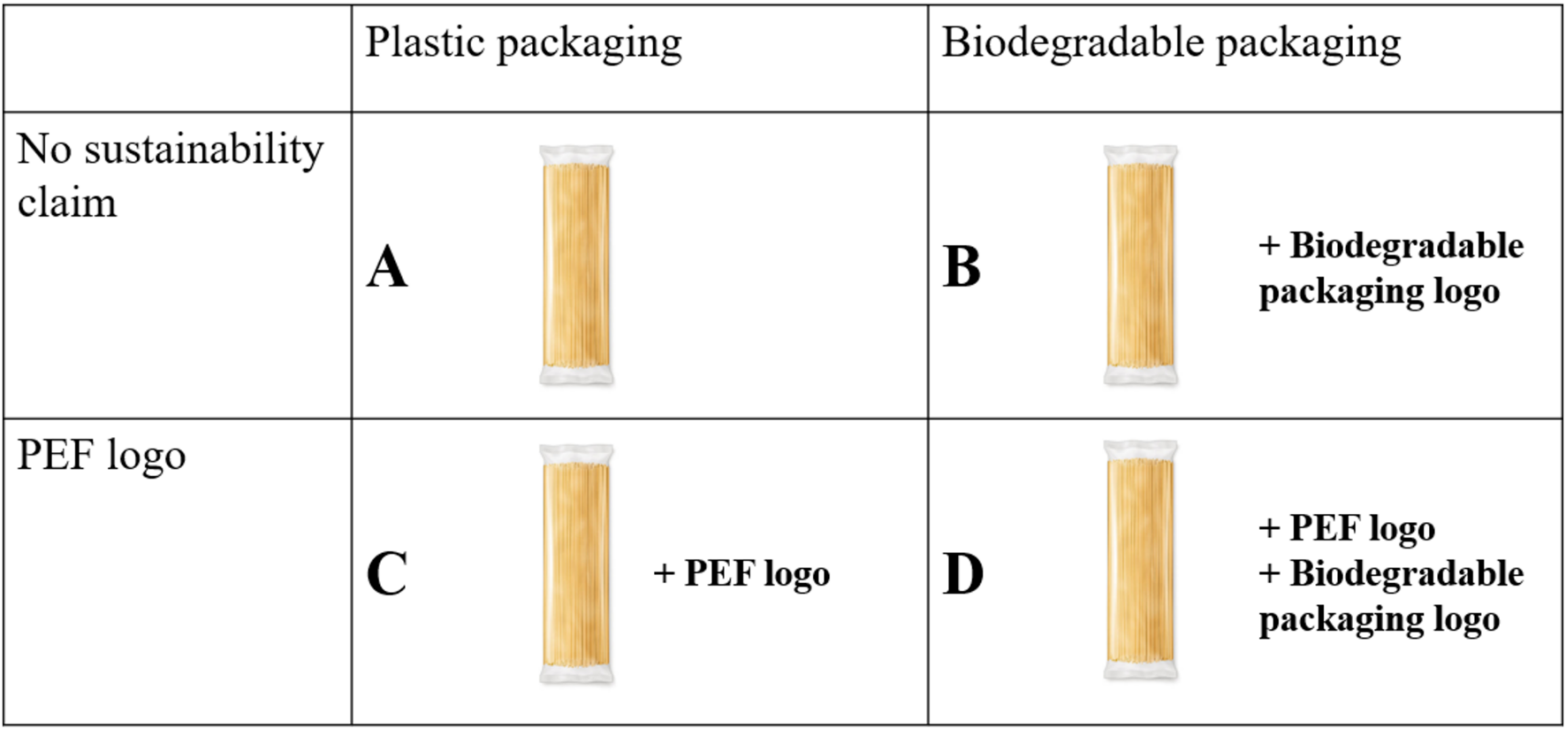
Experimental setup.

We designed an experiment to assess how information about the negative environmental impact of microplastics influences consumers’ willingness to pay (WTP) for four product variants, as shown in Fig. 1. We chose pasta for this experiment because it is a common household ingredient, and a method for calculating Product Environmental Footprint (PEF) is already available for pasta products^30^.

To gather WTP data, we used the Becker-DeGroot-Marschak (BDM) experimental method^31^ for several reasons. Firstly, the BDM method allows us to collect WTP data in a realistic, non-hypothetical setting^32^. Secondly, the BDM method is suitable for field experiments, as it can be conducted one-on-one with participants, offering flexibility in recruitment.

We conducted our field experiment at one of Budapest, Hungary’s largest and most renowned organic farmers’ markets. Farmers’ market attendees often exhibit distinct intrinsic motivations and preferences, providing a valuable context to explore environmentally conscious consumer behavior^33^. By focusing on this unique setting, we aimed to gain insights into the specific dynamics of sustainable purchasing within a community known for its emphasis on organic and environmentally friendly products. Since this market operates only on Saturdays, we collected data over two consecutive market days, February 25 and March 4, 2023. All the products in our study, sourced from certified organic manufacturers, were chosen based on the premise that participants had knowledge that all products were organic, as only certified organic farmers and traders can sell their products at this market. The market primarily takes place in an open-air setting, so our experimental setup mimicked the typical market environment. Weather conditions on the two experimental days were quite similar, minimizing potential environmental effects.

Participants were recruited at an organic market through a combination of leaflet distribution and direct solicitation. Recruiters randomly approached shoppers and inquired about their willingness to participate in an experiment. Potential participants were then screened based on their consumption and purchasing habits of pasta. Recruiters provided only a brief overview of the research to these individuals, intentionally withholding the study’s specific purpose to minimize demand effects. Participants were informed that they would receive a participation fee of 2000 HUF (approximately €5). They were seated in groups of 1–3 people and given detailed explanations of the experimental method. To ensure that all participants understood the BDM experimental method, we conducted a practice run using chocolate bars.

In our study, participants were randomly assigned to one of two groups: the treatment group or the control group. Participants in the treatment group were provided with information regarding the detrimental effects of microplastics on the environment, raising awareness about the issue. In contrast, the control group did not receive this informative content. This between-subjects experimental design allowed us to compare how participants’ knowledge of microplastics’ environmental impact influenced their subsequent behaviors and attitudes.

The four product variants, presented to participants in a randomized order, were available with actual labels on them. Product A served as the benchmark since it is a commercially available organic product in the Hungarian market. Product B was packaged in biodegradable PLA (polylactic acid) packaging. Product C had BOPP (Biaxially Oriented Polypropylene) plastic packaging like Product A but featured a PEF (Product Environmental Footprint) logo on the label, indicating a more sustainable production process than the average pasta product. Product D was packaged in biodegradable material and also included the PEF logo. All four products had a similar appearance, the same label, and the same package size (400 g). The only differences among them were the additional logos indicating the various attributes we were studying.

Both control and treatment groups received the following baseline information on the PEF logo, as it is unknown to consumers, given the fact that PEF logo is not available on food product labels yet: ‘*A Product Environmental Footprint (PEF) is a new method for measuring sustainability performance developed by the European Commission in cooperation with companies and sustainability experts. The aim of the PEF is to improve the validity and comparability of the environmental performance evaluation compared to existing methods. The PEF makes it possible to determine all relevant environmental and health impacts as well as resource-related burdens caused by a product. For the calculation, the entire life cycle of the products is considered.’*

As previously mentioned, participants in the treatment group received extra information on the negative environmental impact of microplastics: *‘It is well known that plastics are now accumulating in the environment, and they can accumulate as microscopic items and even more problematically in the form of microplastic. When they break down, they do not biodegrade, in the sense that they are transformed into carbon dioxide, water, or compost with no ecotoxicity.’*

Following a practice round and an information treatment, physical products were presented to participants one by one in a randomized order. Participants had the opportunity to evaluate the products and examine their labels before providing their willingness-to-pay (WTP) values on a questionnaire. After eliciting the WTP values for all the products, we asked participants about their trust in each product and whether they considered them genuinely organic and sustainable. Trust and perceived sustainability was measured with a single item scale (How much do you trust this product, that it was produced according to the organic standards?; 1—do not trust at all; 7—high trust; How sustainable do you consider this product?; 1—not sustainable at all; 7—very sustainable). We also included questions from the revised New Environmental Paradigm (NEP) scale, which was developed by Dunlap et al.^34^. NEP scale consists of 15 questions about environmental issues, and participants were rating these questions on a 5-point scale (1—strongly disagree; 5—strongly agree).

After the participants filled out the questionnaire, we randomly selected the binding product and randomly picked a price from an urn. The price range was between 300 and 1000 HUF (equivalent to €0.8 and €2.6) in 50 HUF (about 12 cents) increments, based on typical pasta prices in the Hungarian market. If a participant’s willingness to pay (WTP) for the randomly chosen binding product exceeded the randomly drawn price, they would receive the product and the drawn price would be deducted from the participation fee; thus real monetary transaction occurred at the end of the experimental procedure. However, if their bid was equal to or lower than the randomly drawn price, they would not receive the product, and no deduction was required.

Each experimental session took about 15 min to complete. Participants received both the BDM instructions and treatment information verbally, following a written script. The survey was paper-based, and participants completed it themselves.

Our study was registered on Aspredicted.org under number 112970 and obtained ethical approval from the Interim Ethical Committee of the Hungarian University of Agriculture and Life Sciences Doctoral School of Economic and Regional Sciences. All methods were carried out in accordance with relevant guidelines and regulations. Before taking part in the experiment, informed consent was obtained from all participants. Data analysis was carried out using Stata version 17.0.

### Ethical statement

All methods were carried out in accordance with relevant guidelines and regulations. All experimental protocols were approved by the Interim Ethical Committee of the Hungarian University of Agriculture and Life Sciences Doctoral School of Economic and Regional Sciences. Informed consent was obtained from all participants.

## Descriptive statistics

Table 1 displays the socio-demographic characteristics of our sample. We recruited 105 participants who are regular buyers of organic food over two experimental days, with an even distribution between control and treatment groups. The required sample size was determined as 102 participants with a power of 0.8, medium effect size (d = 0.5) and a Type I error rate of 0.05.

**Table 1:** Socio-demographic characteristics of the participants (*n* = 105).

	Control	Treatment	Full sample	*p*-value
	Gender			0.6354
Male	18	20	38	
Female	35	32	67	
	Age group			0.7729
18–25	3	4	7	
26–35	9	4	13	
36–45	5	10	15	
46–55	14	10	24	
56 +	22	24	46	
	Education			0,8140
Elementary	1	0	1	
Vocational	1	0	1	
Highschool	7	13	20	
College	44	39	83	
	Perceived income*			0.8514
Low	8	5	13	
Average	18	26	44	
High	27	21	48	

*Average gross income was 563,500 HUF (appr. 1400 EUR) in 2023 according to the Hungarian Central Statistical Office^36^.

It is worth noting that our sample does not perfectly represent the Hungarian population; it includes an overrepresentation of women over the age 45 with college degree. However, these characteristics align with the socio-demographic profile of regular organic food buyers, as indicated by the Ökobarometer in 2019^35^.

We also gathered information from our respondents about their organic food purchasing habits, sustainability considerations, and motivations for buying organic food. Nearly half of the respondents (46%) reported buying organic food on a weekly basis, with a third purchasing it more frequently. About 18% of respondents bought organic food less often, typically 1–2 times a month. A significant three-quarters of our respondents said they always or often take into account the sustainability and environmental impact of the food they buy.

In terms of motivation, 93% of our participants cited healthiness as their primary motivating factor for buying organic food. Environmental considerations were a motivating factor for 48% of the participants. Additionally, a smaller proportion of respondents, around 24%, were motivated by the better taste of organic food, while 23% were motivated by concerns about animal welfare.

Participants were assigned randomly to either the control or treatment groups. After analyzing the data, we found no significant differences between the two groups concerning gender (t = 0.4756, Pr = 0.6354), age group (t = − 0.2894, Pr = 0.7729), education (t = 0.2359, Pr = 0.8140), and perceived income (t = 0.1878, Pr = 0.8514), all within a 95% confidence interval.

## Results

Table 2 provides an overview of the willingness to pay (WTP) for the four different products used in our experiment, both in the full sample and within the control and treatment groups. Generally, products in plastic packaging without a Product Environmental Footprint (PEF) logo had the lowest WTP value in both the control and treatment groups. Products in plastic packaging with a PEF logo saw an average price premium of 18% across the entire sample. Meanwhile, biodegradable packaging without a PEF logo commanded a 24% price premium compared to pasta in plastic packaging. The combined effect of the PEF logo and biodegradable packaging amounted to a 41% increase in value compared to the benchmark product. Importantly, the measured WTP values did not exhibit statistically significant differences between the control and treatment groups.

**Table 2:** Pasta WTP.

	Full sample (*n* = 105)	Control (*n* = 53)	Treatment (*n* = 52)
Plastic (Product A)	524.6 (285.6)	536.8 (293.5)	512.3 (279.7)
Biodegradable (Product B)	631.9 (319.7)	630.7 (332.4)	633.2 (309.5)
Plastic + PEF (Product C)	619.9 (330.6)	635.8 (336.8)	603.6 (326.6)
Biodegradable + PEF (Product D)	713.1 (387.1)	726.8 (416.8)	699.1 (357.8)

Values are displayed in Hungarian Forint (HUF). 1 HUF is appr. 0.0025 EUR. Mean WTPs are significantly different according to Kolmogorov–Smirnov tests (at *p* < 0.005). Standard deviations are given in brackets.

Table 3 presents the average price premiums calculated on an individual level, with Product A as the benchmark. Participants who received information about the harmful nature of microplastics demonstrated higher price premiums for eco-friendlier products, particularly for Products B and D, which were packaged in biodegradable material. In the case of biodegradable packaging without a PEF logo, the treatment information had a statistically significant effect (t = − 2.0391, Pr = 0.0440) on the price premium, specifically a 31% increase compared to the control group’s 17% price premium. On the other hand, it is worth mentioning that the observed price premium is primarily due to the treatment group’s lower WTP for Product A, rather than a higher WTP for Products B or D. The treatment group, informed about the harmful effects of plastics, reported lower WTP for Products A, C, and D compared to the control group.

**Table 3:** Price premiums.

	Full sample (*n* = 105)	Control (*n* = 53)	Treatment (*n* = 52)
Biodegradable (Product B)	23.8% (35.6)	16.9% (31.1)	30.8% (38.8)
Plastic + PEF (Product C)	18.5% (30)	15.3% (31.4)	21.9% (28.5)
Biodegradable + PEF (Product D)	41.2% (53.9)	36% (56.8)	46.4% (50.8)

Price premium Product B = ((WTPB-WTPA)/WTPA)*100; Price premium Product C = ((WTPC-WTPA)/WTPA)*100; Price premium Product D = ((WTPD-WTPA)/WTPA)*100.

**Figure 2:**
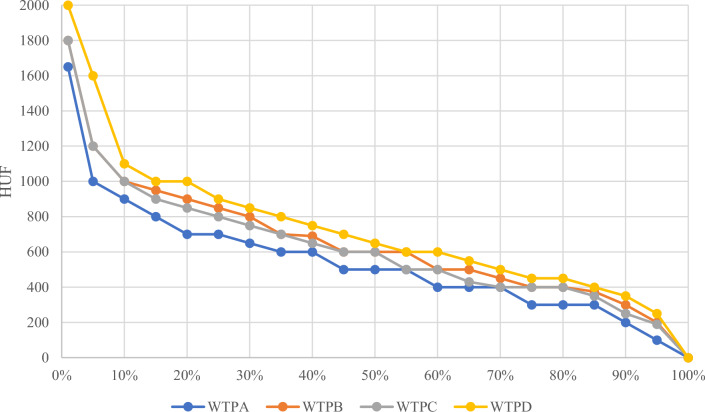
Distribution of the bids of 4 auctioned products.

Figure 2 illustrates the bid distribution in 200 HUF (approximately €0.5) increments. Notably, 10% of the participants were unwilling to pay more than 400 HUF (about €1) for any of the products, while the top 10% of participants were willing to pay over 900 HUF (approximately €2.25). Figure 2 also demonstrates that the WTP distribution for the four different products follows a parallel trend, with no outlier data observed.

**Figure 3:**
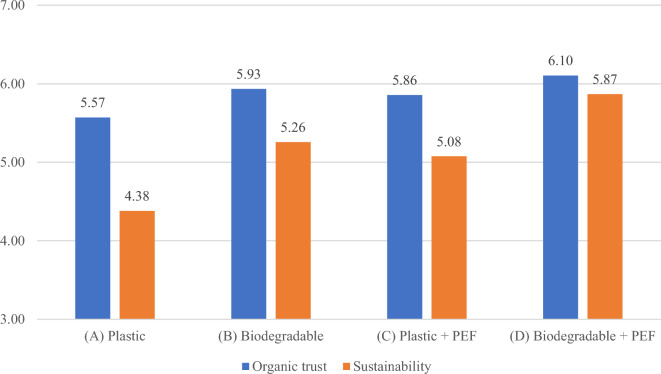
Organic trust and sustainability.

The level of trust and the perceived sustainability of the product exhibits a similar pattern as the WTP values, as shown in Fig. 3. Both trust and sustainability scored the lowest for the product in plastic packaging and reached their highest levels for the product with biodegradable packaging and a PEF logo. The score variance is greater for sustainability compared to organic trust, but no statistically significant differences were observed between the responses of the control and treatment groups.

To uncover correlations between demographics, consumer attitudes, and the proportion of price premiums, we run ordinary least squares (OLS) regressions. The model used for this analysis included the following factors: gender, age, education, income, frequency of organic food purchase, the importance of sustainability, and the New Environmental Paradigm scale.

Our analysis revealed that gender, education, and income significantly influenced price premiums, as it can be observed in Table 4. Female respondents, in general, were willing to pay higher prices for Products B, C, and D. In the control group, female respondents provided significantly higher price premiums for products with a PEF logo. In the treatment group, biodegradable products had significantly higher price premiums among female participants. Age did not play a role in price premiums.

**Table 4:** Preference drivers.

	Full sample (*n* = 105)	Control (*n* = 53)	Treatment (*n* = 52)
Price premium product B			
Gender	1.84*	1.26	2.01**
Age	0.20	− 0.24	0.52
Education	− 1.66*	0.24	− 3.18**
Income	0.79	− 0.54	2.13**
Organic purchase	0.53	0.21	0.84
Sustainability	0.08	− 0.09	0.16
NEP^a^	− 0.06	− 0.51	1.10
Constant	0.69	− 0.03	1.24
*R*^*2*^	0.052	0.043	0.207
*Chi*^*2*^	5.83	2.39	13.61
*p*	0.559	0.935	0.058
Price premium product C			
Gender	2.17**	2.07**	1.41
Age	1.39	0.73	1.12
Education	− 1.28	− 0.35	− 1.89*
Income	0.83	− 0.62	2.14**
Organic purchase	0.67	1.15	− 0.20
Sustainability	0.57	0.65	0.28
NEP^a^	1.23	− 0.01	2.12**
Constant	− 0.37	− 0.96	0.54
*R*^*2*^	0.081	0.136	0.156
*Chi*^*2*^	9.30	8.37	9.60
*p*	0.231	0.301	0.212
Price premium product D			
Gender	2.38**	1.74*	2.34**
Age	0.73	− 0.04	1.23
Education	− 1.08	0.41	− 3.07**
Income	0.91	− 0.56	2.66**
Organic purchase	− 0.35	− 0.00	− 0.19
Sustainability	− 0.05	0.03	− 0.27
NEP^a^	0.26	− 0.69	1.88*
Constant	0.42	− 0.23	1.28
*R*^*2*^	0.061	0.076	0.238
*Chi*^*2*^	6.89	4.35	16.27
*p*	0.441	0.739	0.023

*p < 0.1; **p < 0.05; Cronbach’s alpha values: 0.498^a^.

Breusch–Pagan test of independence: chi^2^ (3) = 150.408, Pr = 0.0000 (full sample); chi^2^ (3) = 66.486, Pr = 0.0000 (control group); chi^2^ (3) = 85.273, Pr = 0.0000 (treatment group).

Significant values are in [bold].

Education level showed an inverse relationship with price premiums among the treatment group respondents, indicating that participants with higher education were less willing to pay a price premium for eco-friendlier products. On the other hand, income had the opposite effect, with higher-income participants showing a greater willingness to pay a higher price premium. However, this effect was only observed in the treatment group, as higher income did not significantly affect price premiums in the control group.

Respondents who expressed concern for environmental issues, as indicated by the New Environmental Paradigm scale, gave higher price premiums for products with a PEF logo among the treatment group respondents. The frequency of organic food purchase and sustainability considerations during food purchase did not significantly impact the price premiums.

## Discussion and conclusions

The research findings point to a general willingness among respondents to pay a premium for environmentally friendly and sustainably produced products, aligning with previous studies^26,37,38^. However, the extent of this price premium is influenced by various factors.

The research findings elucidate noteworthy information treatment effects on consumer willingness to pay (WTP) for products with distinct packaging attributes, particularly under the influence of information regarding the deleterious effects of microplastics. Consistently, products featuring biodegradable packaging and the PEF logo commanded the highest WTP, signifying a discernible consumer preference for environmentally conscious choices. Concurrently, traditional plastic packaging elicited the lowest prices, indicative of a discernible market shift toward sustainability.

An in-depth analysis of the treatment group unveils a significant revelation. Despite the recognized sustainability symbol in the form of the PEF logo, the introduction of targeted information highlighting the adverse effects of microplastics was observed to potentially influence consumer perceptions of biodegradable packaging pricing. This underscores the potent impact of focused knowledge dissemination, even in the presence of established sustainability markers.

Drawing parallels with the findings of Steenis et al.^24^, our results align with the notion that informed consumers are inclined to pay a premium for products with eco-friendly packaging. The discussion on the information treatment effect, particularly concerning the repercussions of microplastics, emphasizes the potential role of information in shaping consumer perceptions.

Moreover, the nuanced response observed across demographic segments adds complexity to our understanding. Female respondents exhibited a significantly higher WTP for two distinct environmentally conscious attributes: biodegradable packaging and products featuring the PEF logo. This finding underscores the effectiveness of general information dissemination about the harmful effects of microplastics, as emphasized by Van Asselt et al.^28^. The presence of the PEF logo particularly resonated with female consumers, suggesting a heightened awareness and concern about microplastic pollution. This awareness likely influenced their bidding behavior, reflecting a proactive preference for products perceived to have lower environmental impact.

Nevertheless, the variability in the information treatment effect across diverse consumer groups introduces a layer of intricacy. In the treatment group, factors such as education level, perceived income, and environmentally friendly behavior emerged as influential in shaping how respondents processed negative information about microplastics. This interplay suggests the necessity for tailored communication strategies to maximize the impact of sustainability information across heterogeneous consumer profiles.

Interestingly, respondents with higher education levels were willing to pay a smaller price premium for environmentally friendly products, indicating that information treatment was less effective for them. It is also possible that more educated consumers might already possess greater knowledge about microplastics and eco-labels in general. Conversely, lower-educated respondents were willing to pay a higher price premium, suggesting that educational efforts were more effective for this group. Higher perceived income often correlates with a higher willingness to pay^38^, as observed in this study, but only in the treatment group, indicating that information treatment had a positive effect on the willingness to pay of higher-income individuals. Similarly, those who identified as environmentally conscious were significantly more willing to pay a higher price for environmentally friendly products when provided with information. Therefore, even respondents with higher NEP scores needed encouragement to pay a higher price for products with biodegradable packaging and the PEF logo.

The presence of the PEF logo alone was unable to achieve a higher price premium compared to products with only biodegradable packaging, despite signaling higher sustainability expectations. A potential reason for the lack of a significant premium generated by the PEF might also be that respondents already perceive organic food to generally be more environmentally friendly. However, it is evident that when biodegradable packaging and the PEF logo were used together, a higher willingness to pay was achieved among respondents compared to when they were used separately. One possible explanation is that the PEF logo considers sustainability metrics that consumers cannot easily verify while shopping, making it a credibility attribute. This credibility is further supported by the environmental-friendliness of the packaging, which is easily recognizable by consumers, reinforcing trust in the PEF logo and leading to a higher willingness to pay when both biodegradable packaging and the PEF logo are used together.

A similar situation was observed with organic trust and perceived sustainability. Biodegradable packaging significantly increased trust in the product’s organic nature and perceived sustainability more than a product marked only with the PEF logo, but the combination of both achieved the highest value among the four products.

In conclusion, our study contributes insights into the influence of information on WTP, with a specific emphasis on information treatment effects related to the harmful effects of microplastics. These findings carry significant policy implications, highlighting the imperative for targeted communication strategies to effectively convey the environmental consequences of product choices. As consumer awareness expands, policymakers can leverage these insights to formulate initiatives that not only promote sustainable practices but also harness the power of information to induce positive behavioral change in the marketplace.

The presence of environmentally friendly packaging and the PEF logo has a positive impact on both willingness to pay and consumer trust in the product’s sustainability. Despite its holistic approach, the PEF logo does not increase the price premium as much as biodegradable packaging alone, but when used together, it seems to instill greater consumer trust that leads to a higher willingness to pay for a given product.

The information treatments about the harmful effects of microplastics were not effective for all consumer groups. However, for female, higher-income, and more environmentally conscious respondents, a significant increase in willingness to pay was observed. Therefore, it can be concluded that it may be worthwhile to share such information with these consumer groups. Unfortunately, for those who consider themselves less environmentally conscious, negative information treatment was less effective, making it difficult to reach the very group that should be encouraged to make more environmentally friendly purchasing decisions.

In light of the research findings, this paper recommends a dual strategy. Prioritizing biodegradable packaging, particularly when accompanied by the PEF logo, can be crucial for enhancing consumer trust and willingness to pay. Additionally, tailoring dissemination of information on microplastics to specific demographics would be essential for optimal effectiveness.

It is important to note the limitations of this study. The research sample comprises a narrow, specialized consumer group—regular purchasers of organic food products. Thus, results might not be generalizable to the general food consumers. Additionally, even in situations involving no uncertainty, such as with regular products, the BDM mechanism may not be incentive compatible^39^. Nevertheless, our information treatment effect should be valid given the random assignment to both treatment and control groups. The study also focused solely on one type of product, pasta, so different factors may come into play when considering environmentally friendly purchasing habits for other product categories. Future research should explore the robustness of our findings by including broader consumer groups and considering a wider range of products.

## Data Availability

The datasets generated and/or analyzed during the current study are available from the corresponding author upon reasonable request.
